# Strand-specific transcriptomes of Enterohemorrhagic *Escherichia coli* in response to interactions with ground beef microbiota: interactions between microorganisms in raw meat

**DOI:** 10.1186/s12864-017-3957-2

**Published:** 2017-08-03

**Authors:** Wessam Galia, Francoise Leriche, Stéphane Cruveiller, Cindy Garnier, Vincent Navratil, Audrey Dubost, Stéphanie Blanquet-Diot, Delphine Thevenot-Sergentet

**Affiliations:** 1 UMR 5557 Ecologie Microbienne, Research Group on Bacterial Opportunistic Pathogens and Environment, CNRS, VetAgro Sup and Université de Lyon, Lyon, France; 2 Université Clermont Auvergne, INRA, UMRF, F-15000 Aurillac, France; 3 UMR UCA INRA 454 MEDIS Microbiota Digestive environment and Health, Université Clermont Auvergne, 63000 Clermont-Ferrand, France; 4 VetAgro Sup, Campus Agronomique de Lempdes, Lempdes, France; 5 Alternative Energies and Atomic Energy Commission (CEA), Genomic Institute Genoscope & CNRS-UMR8030 & Evry University, Laboratory of Bioinformatics Analysis in Genomics and Metabolism, Evry, France; 6 PRABI, Rhône Alpes Bioinformatics Center, UCBL, Lyon1, Université de Lyon, Lyon, France; 7 UMR 5557 Ecologie Microbienne, CNRS, Université de Lyon, Lyon, France; 8 Reference Laboratory for Escherichia coli including Shiga Toxin-Producing E. coli, VetAgro Sup, Campus Vétérinaire de Lyon, Université de Lyon, Marcy l’Etoile, Lyon, France

**Keywords:** RNA-Seq, EHEC, Ground beef, Natural microbiota, 16S metagenomics analysis

## Abstract

**Background:**

Enterohemorrhagic *Escherichia coli* (EHEC) are zoonotic agents associated with outbreaks worldwide. Growth of EHEC strains in ground beef could be inhibited by background microbiota that is present initially at levels greater than that of the pathogen *E. coli.* However, how the microbiota outcompetes the pathogenic bacteria is unknown. Our objective was to identify metabolic pathways of EHEC that were altered by natural microbiota in order to improve our understanding of the mechanisms controlling the growth and survival of EHECs in ground beef.

**Results:**

Based on 16S metagenomics analysis, we identified the microbial community structure in our beef samples which was an essential preliminary for subtractively analyzing the gene expression of the EHEC strains. Then, we applied strand-specific RNA-seq to investigate the effects of this microbiota on the global gene expression of EHEC O26_21765_ and O157_EDL933_ strains by comparison with their behavior in beef meat without microbiota. In strain O26_21765_, the expression of genes connected with nitrate metabolism and nitrite detoxification, DNA repair**,** iron and nickel acquisition and carbohydrate metabolism, and numerous genes involved in amino acid metabolism were down-regulated. Further, the observed repression of *ftsL* and *murF*, involved respectively in building the cytokinetic ring apparatus and in synthesizing the cytoplasmic precursor of cell wall peptidoglycan, might help to explain the microbiota’s inhibitory effect on EHECs. For strain O157_EDL933_, the induced expression of the genes implicated in detoxification and the general stress response and the repressed expression of the *peR* gene, a gene negatively associated with the virulence phenotype, might be linked to the survival and virulence of O157:H7 in ground beef with microbiota.

**Conclusion:**

In the present study, we show how RNA-Seq coupled with a 16S metagenomics analysis can be used to identify the effects of a complex microbial community on relevant functions of an individual microbe within it. These findings add to our understanding of the behavior of EHECs in ground beef. By measuring transcriptional responses of EHEC, we could identify putative targets which may be useful to develop new strategies to limit their shedding in ground meat thus reducing the risk of human illnesses.

**Electronic supplementary material:**

The online version of this article (doi:10.1186/s12864-017-3957-2) contains supplementary material, which is available to authorized users.

## Background

Bacteria rapidly program their gene expression to survive changing environments and resist challenging conditions. Such adaptation through gene expression can radically change bacterial physiology and pathogenicity. RNA-sequencing has revolutionized the study of gene expression because, in addition to quantifying transcriptional output, it allows the detection and characterization of all transcripts in a genome. This innovative, non a priori tool is rapidly becoming the method of choice for revealing new functional genes and pathways in individual microbes [1, 2] as well as in complex environmental communities, e.g. from the sea [3, 4] and the human gut [5, 6]. However, deciphering the behavior of one population within a complex microbiota comprising related taxonomic species is a real challenge.

EHECs are food-borne zoonotic agents associated with disease outbreaks worldwide and represent a serious public health concern. They are strongly associated with severe forms of infection such as hemorrhagic colitis and, in some extreme cases, hemolytic-uremic syndrome (HUS) [7, 8]. Human infection is typically acquired through ingestion of contaminated food (undercooked ground beef, dairy products, vegetables, etc.) and water. In 2014, 5955 confirmed cases of EHEC infection were reported in the EU. That year as in previous years, the most commonly reported EHEC serogroup was O157 (46.3% of cases with known serogroup), followed by serogroups O26, O103, O145, O91, O146 and O111 (EFSA, Zoonosis 2014). Beef cattle are the primary reservoir of EHECs [9] which they carry in and excrete from their gastrointestinal tract. These animals stay without any symptoms of disease [10]. Cattle faeces is considered the main source of EHEC contamination of carcasses during slaughter [11].

Data from EFSA (Zoonosis, 2014) [12] show that the highest proportion of STEC-positive samples were reported from ruminant meat (goat, sheep, cattle and deer). Over the same period, STEC were reported in about 1% of cheese samples, particularly sheep and goat milk cheeses, while contamination was rare in RTE (Ready-To-Eat) food of plant origin. In France, the majority of STEC infections point to the consumption of undercooked minced meat [13]. We hypothesized that biotic and abiotic factors in meat could have an impact on the growth, physiology and virulence of EHEC strains. The meat microbiota might antagonize human pathogens by many different mechanisms, including production of compounds with antimicrobial activity as well as competition for attachment sites or nutrients [14, 15]. To date, few data about the interaction between bovine meat microflora and EHECs are available.

The aim of this study was to develop a new RNA-seq tool for use with polymicrobial samples, among others. We identified metabolic pathways of EHEC that were altered by natural microbiota, to improve our understanding of the mechanisms controlling the growth and survival of EHECs in ground beef. We first conducted a 16S metagenomics analysis to identify the natural microbiota present in some ground meat. We then used strand-specific RNA-seq to investigate the effects of the ground beef background microbiota on the overall gene expression of EHEC strains O2621765 and O157EDL933 by comparison with their behavior in beef meat without microbiota.

## Methods

### Bacterial strains

The EHECs used in this study were 0157:H7 strain EDL933 (O157_EDL933_) and O26:H11 strain 21,765 (O26_21765_), isolated from Michigan ground beef and human fecal samples respectively and linked to human EHEC infections [16, 17].

### Sample preparation

A single beef piece of about 11 kg taken from a local slaughterhouse was kept 72 h at 4 °C before processing in our laboratory. The outer part of the muscle was aseptically separated from the inner part, considering the first to be highly contaminated and the second to be devoid of bacteria. Each part was aseptically minced and divided into 4 portions of 200 g before homogenization in a sterile stomacher bag (Spiral Biotech, Bethesda, Md.). For each trial, the ground beef was checked for the absence of *E. coli* O26:- and O157:- [18].

Frozen cultures of EHEC were revived on plate-count agar (PCA, BioMérieux, Marcy l’Etoile, France) by incubation at 37 °C for 24 h. One colony of each strain was selected and cultured in BHI (Brain Heart Infusion) overnight at 37 °C without agitation. Cells were harvested by centrifugation at 4000×g for 15 min at 12 °C. Pellets were suspended in 0.1% peptone (Oxoid) water (12 °C).

The ground beef was then inoculated as follows: each 200 g portion was inoculated with 20 ml (10^8^ CFU/ml) of EHEC strains O157_EDL933_ or O26_21765_ to obtain a final concentration of about 10^7^ CFU/g. For the control experiment, a similar amount of sterile peptone water was added. Each ground beef sample was hand mixed to distribute the inoculum evenly. Samples were then incubated in a sterile stomacher bag without agitation at 12 °C for 7 days. This temperature (12 °C) was chosen as being sufficient for identifying inter-organism interactions without halting EHEC growth [19]. Three biological replicates were performed for each condition.

### Ground beef microbiota and EHEC enumeration

After serial dilutions of the samples stomached 1 min in BPW (Buffered Peptone Water, bioMerieux, Marcy l’Etoile, France), decimal dilutions of the samples were plated and incubated in appropriate conditions: for aerobic bacteria, soy agar plates were incubated at 37 °C for 24 h; for LAB, MRS (deMan Rogosa Sharpe; Difco) agar plates were incubated in a GasPak jar (BBL, Becton Dickinson Microbiology Systems) at 30 °C for 48 h, complemented with a gas generating sachet (BD GasPak EZ Gas Generating Sachet). For enumeration of O157_EDL933_ and O26_21765_ strains, dilutions were spread respectively onto ChromID O157:H7 agar (ChromID O157:H7 + 0.05 mg/l cefixime +2.5 mg/l tellurite, bioMérieux) and chromogen agar *E. coli* Brillance. The plates were incubated at 37 °C for 24 h. All counts were performed in duplicate.

### Collection of EHEC cells and ground beef microbiota for metagenomic and transcriptomic analysis

Beef samples were diluted (1:4 *w*/*v*) in cold BPW containing 0.1% (*v*/v) Tween X-20 and pummeled for 1 min using a stomacher lab blender (Seward Medical, London, UK). Following pummeling, the filtered samples were clarified by centrifugation at 200 g for 5 min at 4 °C. The supernatants were removed to fresh tubes, and cells were collected by centrifugation at 3500 g for 10 min at 4 °C.

### Metagenomic analysis

#### Genomic DNA extraction, PCR amplification and amplicon sequencing

DNA was extracted from samples using a Genomic DNA kit (NucleoSpin Tissue, Germany). To minimize PCR bias, DNA extracts from the triplicate samples were pooled and used as the template DNA. DNA purity (A260/A280 and A260/A230) and quantity were measured with a NanoDrop ND-1000 spectrophotometer (Thermo Scientific). The V1-V3 region of the 16S rRNA gene was amplified by PCR using the primers 27F (5′-AGAGTTTGATYMTGGCTCAG-3′) and 519R (5′-GWATTACCGCGGCKGCTG-3′) [20]. The libraries were prepared with Nextera XT version 3 chemistry (Illumina) and the Illumina MiSeq platform sequencer produced 300 bp paired-end reads.

#### Quality trimming, taxonomic assignments, and operational taxonomic unit clustering

**Fig. 1:**
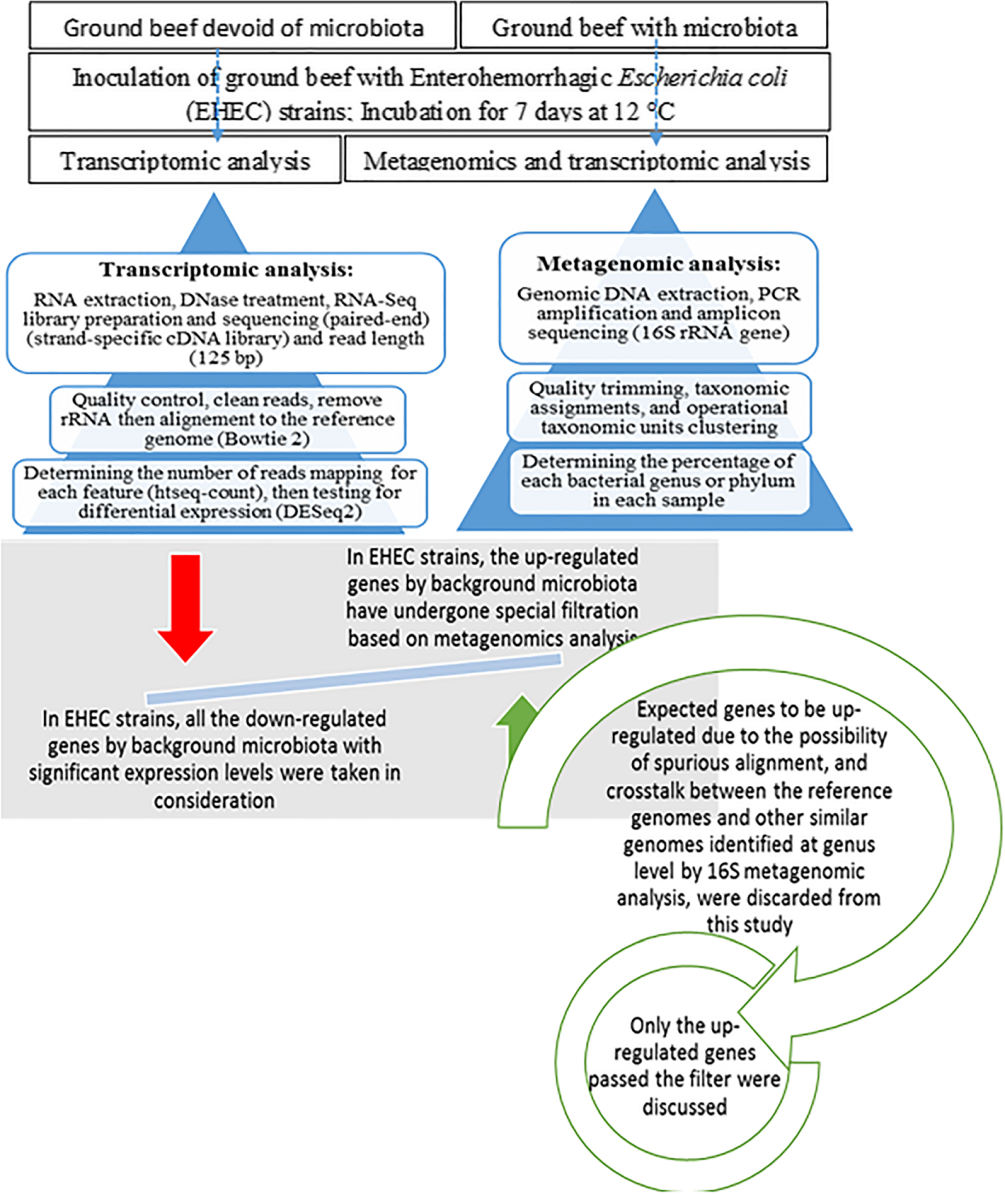
Workflow for studying the overall gene expression of Enterohemorrhagic *Escherichia coli* (EHEC) strains O157:H7 EDL933, O26:H11 21,765 in ground meat with or without background microbiota

The paired-end sequences were assembled into a single sequence, ca. 510-bp. The sequences were trimmed using MOTHUR software [21] to remove primers, long homo-polymers and sequences shorter than 100 bases (Fig. 1). Reads with ambiguous bases were removed from the data set. Sequence alignment, chimeras checking, distance calculation, OTU (Operational Taxonomic Unit) clustering and selection of non-redundant sequences were performed using MOTHUR version 1.34.4 [21]. The SILVA reference file for bacteria [22] was used to align the sequences. Indeed, to assign our sequence reads to the prokaryotic taxonomy, the Naïve Bayesian Classifier tool hosted by Ribosomal Database Project (RDP Classifier) [23] and described by Wang et al. (2007) was used [24]. This method runs a bootstrapping algorithm to find the confidence limit of the assignment of each query. A bootstrap cutoff value of 0.6 was used in our study. The percentage of each bacterial genus or phylum was calculated individually for each sample, providing information on relative abundance within the individual samples based on the relative numbers of reads in each one. Sequencing reads are available at the European Nucleotide Archive under study number PRJEB13580 (ERS1119543-ERS1119545).

### RNA sequencing analysis

#### RNA extraction and DNase treatment

Briefly, cell pellets were re-suspended in 10 mL of TRIzol Reagent (Ambion) and incubated for 5 min at 20 °C. One volume of chloroform was added to 5 volumes of homogenate; nucleic acids were then separated from proteins by centrifugation at 12,000 *g* for 15 min at 4 °C. One volume of the aqueous supernatant was mixed with 1 volume of ethanol 70%. RNA extractions were then performed according to the Qiagen recommendations, including on-column RNA purification (RNeasy Midi Kit, QIAGEN).

#### DNase treatment

The TURBO DNA-free kit (Ambion) was used for the DNase treatment. Total RNA was treated using a rigorous protocol that includes a double quantity of DNase (6 units). Reactions were terminated with the addition of the DNase inactivation reagent (0.2 × the reaction volume) according to the kit instructions. The presence of DNA contamination was assessed by real time PCR targeting of the *eae* gene [25]. RNA integrity (RIN) values [26] were determined by running 1 μl aliquots on a Bioanalyzer 2100 (Agilent, Technologies, Inc.) resulting in clear patterns with prominent 16S and 23S ribosomal bands and RIN values of ≥8.

#### RNA-Seq library preparation and sequencing

Library construction and sequencing were performed at the MGX-Montpellier GenomiX platform. Ribo-Zero rRNA Removal Kit Bacteria (Illumina, San Diego, CA) was used to remove ribosomal RNA from 2 μg of total RNA. For each sample, 100 ng mRNA-enriched fraction was used to construct sequencing libraries using Illumina’s TruSeq Stranded mRNA Sample Prep Kit (Low throughput). The mRNAs were fragmented into small pieces using divalent cations under elevated temperature. The cleaved RNA fragments were copied into first strand cDNA using SuperScript II reverse transcriptase, Actinomycin D and random hexamer primers. The addition of Actinomycin D prevents spurious DNA-dependent synthesis, while allowing RNA-dependent synthesis, improving strand specificity. The second strand cDNA was synthesized by replacing dTTP with dUTP. The incorporation of dUTP quenches the second strand during amplification, ensuring that only the first cDNA strand is efficiently amplified [27, 28]. These cDNA fragments then had the addition of a single ‘A’ base and subsequent ligation of the adapter. The products were then purified and enriched with 15 cycles of PCR. The final cDNA libraries were validated with a DNA 1000 Labchip on a Bioanalyzer (Agilent) and quantified with a KAPA qPCR kit. For each sequencing lane of a flowcell V4, three to six libraries were pooled in equal proportions, denatured with NaOH and diluted to 7.5 pM or 8 pM before clustering. Cluster formation, primer hybridization and pair end-read 125 cycles sequencing were performed on cBot and HiSeq2500 (Illumina, San Diego, CA) respectively. Sequencing reads are available at the European Nucleotide Archive under study number PRJEB13600 (ERS1127228-ERS1127233; ERS1127235-ERS1127239; ERS1138027).

#### RNA-Seq data processing

The raw reads of RNA-seq data were processed using the galaxy.prabi.fr web service and the computing facilities of the LBBE/PRABI (Fig. 1) [29–31]. To clean the sequences, the adapters that were ligated to the 5′ or 3′ end with a maximum allowed error rate of 0.1 and a minimum overlap length of 3 bp were removed using Cutadapt (version 1.6) [32]. Low quality sequences were then removed using Trimmomatic (version 0.32.1) [33] with the following explicit parameters: paired-end data: the minimum quality required to keep a base was 20 and the reads were trimmed when the average quality over a 4 bp window dropped below 22, starting from the 5′ end of the read. Moreover, trimmed/clipped sequences were kept only if they were at least 30 bp long. The reads were then aligned and assigned to the reference genome using the Bowtie 2 package (version 0.2) [34], with the following parameters: paired-end alignment modes with a maximum fragment length for valid paired-end alignments of 500 bp, and end-to-end read alignment. We then used the mapped files to run HTSeq-count (version 0.4.1) [35]. This script takes an alignment file in BAM format and a feature file in GFF format and calculates the number of reads mapping to each feature. The paired-end strand-specific RNA-Seq reads were assigned to these features based on their overlapping genomic coordinates and strand orientation. Reads with more than one reported alignment were not counted for any feature. Where the reads overlapped more than one feature, the intersection-nonempty mode was used. Counts of RNA-Seq fragments were computed for each coding DNA sequence (CDS) according to the genome annotations provided by the MicroScope platform (https://www.genoscope.cns.fr/agc/microscope/) (*E. coli* O26:H11 21,765: chromosome ECO26H.gff, plasmid ECO26H_p.gff; *E. coli* O157:H7 EDL933: chromosome NC_002655.gff, plasmid AF074613.gff) [16, 36]. Then the count tables generated by the HTSeq count were used as input to run DESeq2 (version 2.1.6.0) [37]. This tests for differential expression based on a model using the negative binomial distribution. The DESeq2 package provides its own normalization approach. The read count K_ij_ for gene i in sample j is described with a generalized linear model (GLM). DESeq2 models read counts Kij with mean μ_ij_ and dispersion α_i_. The mean is taken as a quantity q_ij_, proportional to the concentration of cDNA fragments from the gene in the sample, scaled by a correction factor s_ij_. To estimate these factors, the DESeq2 package uses the median-of-ratios method [38]. The principal idea is that non differential expression (DE) genes should have similar read counts across samples, leading to a ratio of 1. Supposing most genes are not DE, the median of this ratio for the sample provides an estimate of the normalization factor that should be accounted for differences in sequencing depth between samples. This method is advantageous to calculate gene-specific normalization factors s_ij_ to account for further sources of technical biases such as differing dependence on GC content or gene length. The False Discovery Rate (FDR) was controlled by adjusting *p*-values with the Benjamini Hochberg method [39]. In this report, only genes showing a ≥ 2 fold up-regulation or down-regulation with a minimum normalized read count = 10 and a Benjamini-Hochberg FDR adjusted *p*-value of ≤0.005 were considered to be differentially regulated. The transcript lists resulting from DESeq2 were then analyzed using the MicroScope platform, a web-based framework for functional analysis of large numbers of genes [40].

## Results and discussion

### Microbial community structures in raw ground beef inoculated or not with strains O26_21765_ or O157_EDL933_

Our experiment was designed so that EHEC strains would have sufficient time to adapt to the beef environment and that we could retrieve sufficient RNA(s) for analysis. A large initial EHEC inoculum (3 log greater than the natural microbiota counts) was chosen to ensure that the meat microbiota, present at an initial level of about 10^4^ CFU/g, would not impair the growth of either O157_EDL933_ or O26_21765_ [19]. As expected, after 7 days of incubation, no flora were detected in the inner part of the muscle, used as control, whereas in the outer part about 8 log CFU/g of each type of bacteria (aerobic and LAB) were counted (Additional file 1: Table S1). In the non-inoculated ground meat samples (outer part of the muscle) commensal *E. coli* counts were about 3 logs below the level of the other natural microbiota species. In artificially contaminated minced meat obtained from the outer part, after 7 days’ incubation at 12 °C the commensal *E. coli* were no longer detectable. They had probably lost out in competition with the two EHEC strains (O157_EDL933_ and O26_21765_) inoculated at an initial level of about 10^7^ CFU/g. The counts of both these EHEC strains were 1 log higher than the natural microbiota counts. The presence of the meat microbiota at an initial level of about 10^4^ CFU/g did not impair the growth of either O157_EDL933_ or O26_21765_ (See Additional file 2: Table S2).

**Fig. 2:**
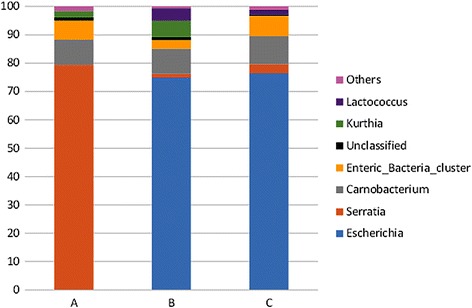
Genus level distribution of sequences based on the 16S rRNA gene libraries constructed from a ground beef sample with natural microflora (**a**) and ground beef samples inoculated with *E. coli* O26:H11 21,765 (**b**) or *E. coli* O157:H7 EDL933 (**c**) strains. Only taxa with a percentages above 1% are displayed in the histograms

Using 16S metagenomics analysis enabled us to characterize the bacterial populations of our samples and evaluate their relative abundances after 7 days of incubation at 12 °C. A total of 2,852,952 bacterial 16S ribosomal RNA sequences, obtained from 3 samples (ground beef with natural microbiota and ground beef inoculated with strain O26_21765_ or strain O157_EDL933_) were analyzed. At the genus level, 2,827,664 sequences were classified (bootstrap value cutoff = 0.6). However, 0.9% of total sequences were either unclassified or classified at a higher taxonomic level. In the natural microbiota of the ground beef samples, our data clearly showed that *Serratia* dominated (79.4% incidence). Sequences belonging to the genus *Carnobacterium* and the enteric bacteria cluster amounted to 8.8% and 6.8% respectively. *Carnobacterium* is a psychrotrophic bacterium frequently isolated from foods, including meat [41]. Sequences from *Kurthia* and other bacteria were represented in smaller proportions in the control samples. In experimentally contaminated samples, *Escherichia* dominated the microbiota (74.9% for O26_21765_ and 76.4% for O157_EDL933_) and outnumbered *Serratia*, whose sequences were detected in less than 5% (Fig. 2; Additional file 3: Table S3). The number of sequences belonging to the genus *Carnobacterium* was not affected by the EHEC strains.

The analysis of the microbial composition of our samples is not discussed any further in this study. For our purpose, identification of the microbial community structure at the genus level was an essential preliminary for subtractively analyzing the gene expression of the EHEC strains. It was required to identify the genes of EHEC strains expected to be differentially expressed due to the possibility of spurious alignment and crosstalk between the EHEC genes and any other similar gene identified as being part of genome sequencing data provided by GenBank and corresponding to the most abundant genus identified by 16S metagenomics analysis (Fig. 1).

### Global gene expression of EHEC strains O26_21765_ and O157_EDL933_ in ground meat with or without background microbiota

We set out to investigate the effects of ground beef background microbiota on the overall gene expression of EHEC strains O26_21765_ and O157_EDL933_, by comparison with their behavior in beef without microbiota. Even if eukaryotic rRNA acquired from bovine tissue cells could not be depleted owing to partial degradation, we were able to detect differential expression of numerous genes with high statistical significance thanks to the biological replicates and robust bacterial rRNA depletion. Bacterial rRNA depletion was nearly complete in all samples, since fewer than 2.3% of all high quality reads were aligned with bacterial rRNA-encoding genes (See Additional file 4: Table S4). Haas et al. (2012) has also claimed that changes in gene expression could be analyzed by RNA-Seq data analysis when the number of fragments per sample is reduced to 2–3 million [42].

Sequencing depths of 3.8 ± 1.5 and 4.3 ± 2.1 million gene mapped fragments were obtained in the O26_21765_ and O157_EDL933_ datasets respectively (Additional file 4: Table S4). Based on mean normalized counts of gene-mapped fragments, the numbers of ORFs with at least 10 fragments that DESeq2 detected were 5035 for O157_EDL933_ and 4865 for O26_21765_, amounting to 88.1% and 86.7% of their annotated genes respectively.

**Fig. 3:**
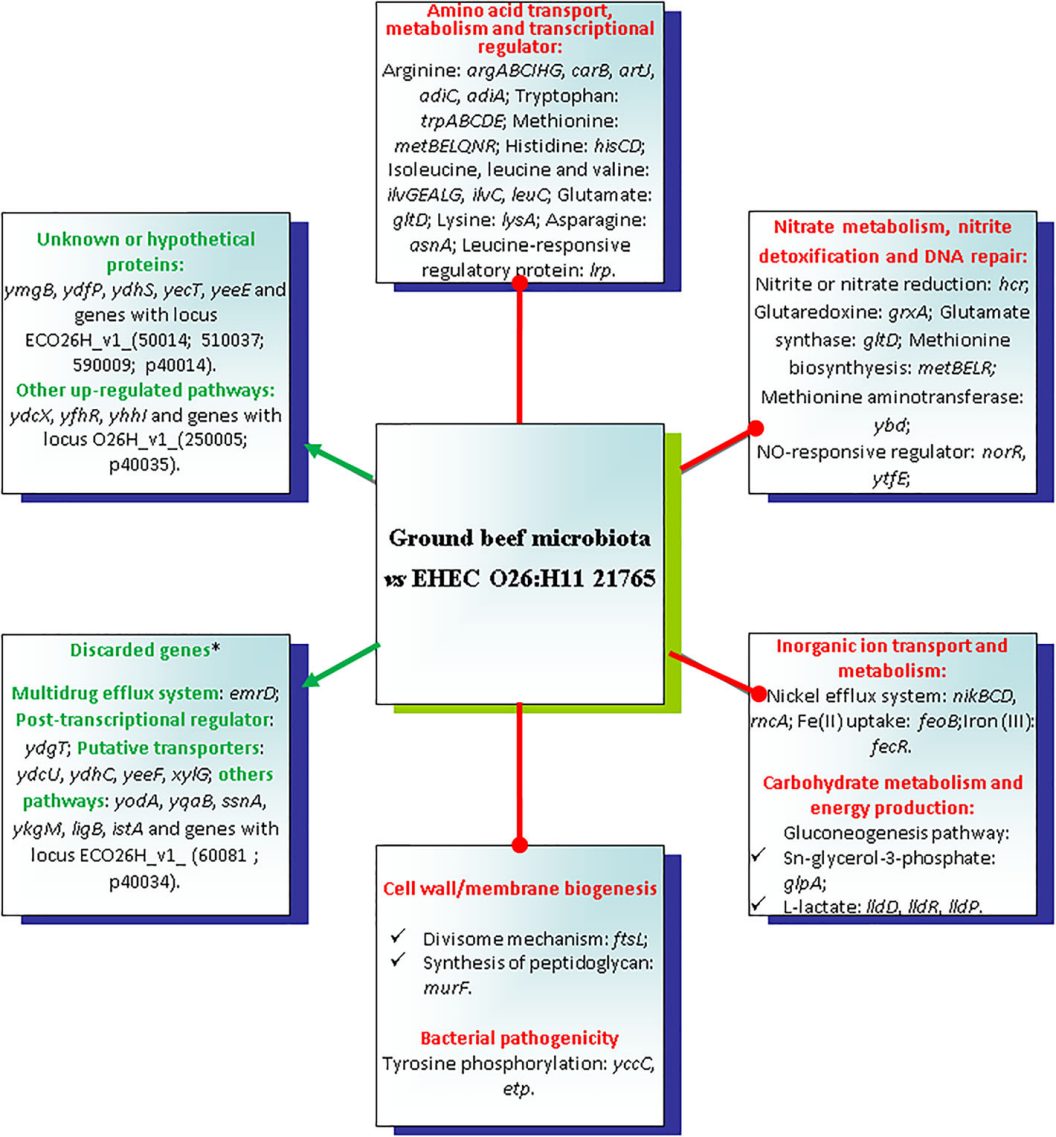
Summary of the main pathways and regulations affected by background microbiota in Enterohemorrhagic *Escherichia coli* O26:H11 strain 21,765 in ground beef. Green arrows with head indicate up-regulation and red arrows without head down-regulation. * Genes expected to be up-regulated owing to the possibility of spurious alignment and crosstalk between the reference genome and other similar genomes identified at genus level by 16S meta-genomic analysis, were discarded from the study (For discarded genes see Additional file 6: Table S6)

**Fig. 4:**
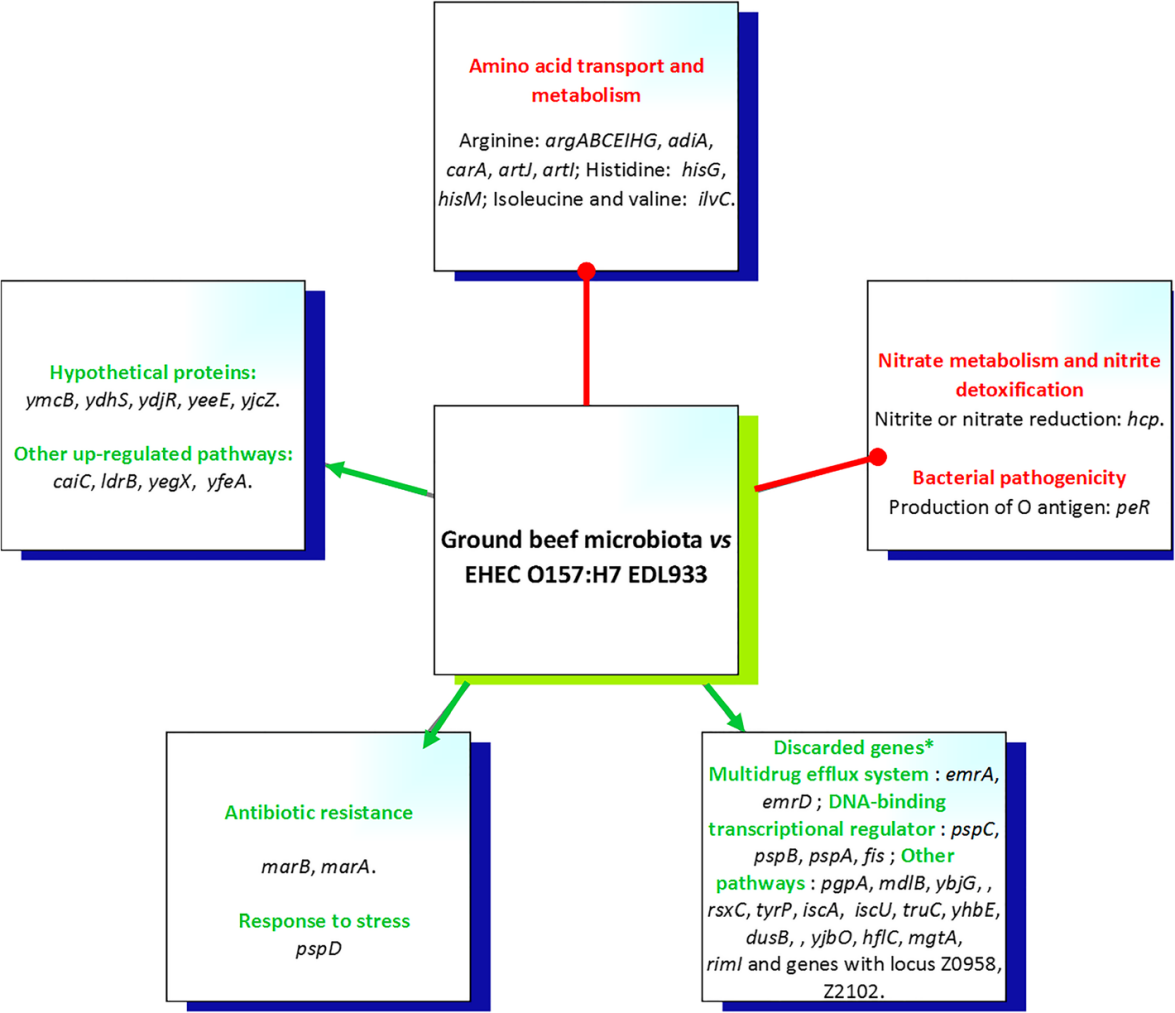
Summary of the main pathways and regulations affected by background microbiota in Enterohemorrhagic *Escherichia coli* O157:H7 strain EDL933 in ground beef. Green arrows with head indicate up-regulation and red arrows without head down-regulation. * Genes expected to be up-regulated owing to the possibility of spurious alignment and crosstalk between the reference genome and other similar genomes identified at genus level by 16S meta-genomic analysis, were discarded from the study (For discarded genes see Additional file 8: Table S8)

Analysis of gene expression with significant expression levels *≥*2-fold and adjusted *P*-value *≤*0.005 revealed that the majority of genes did not differ significantly between conditions. Strain O26_21765_ had a large number of significantly changed genes than O157_EDL933_. Overall, of 5611 coding sequences of strain O26_21765_, 95 were expressed at significantly lower levels in samples with microbiota compared to those without microbiota, and 28 were significantly up-regulated (Fig. 3; Tables 1 and 3; Additional file 5: Table S5 and Additional file 6: Table S6). The corresponding numbers for down- and up-regulated genes in strain O157_EDL933_ were 21 and 25 respectively (Fig. 4; Tables 2 and 4; Additional file 7: Table S7 and Additional file 8: Table S8).

**Table 1:** Genes significantly down-regulated in *Escherichia coli* O26:H11 21,765 in samples with microbiota compared to those without microbiota

Locus (ECO26H_v1_ #)	Gene name	Mean of normalized counts	FC^a^	adj. *p* ^b^	Function or product
*Nitrate metabolism and nitrite detoxification*					
180,052	*hcr*	90	−2.3	0.003	HCP oxidoreductase, NADH-dependent
110,034	*ybdL*	1542	−2.1	3.3E-05	Methionine aminotransferase, PLP-dependent
180,029	*grxA*	180	−2.1	0.0006	Glutaredoxin 1, redox coenzyme for ribonucleotide reductase (RNR1a)
520,050	*norR*	126	−2.0	0.0002	NO-responsive regulator
790008	*ytfE*	47	−2.3	0.005	Regulator of cell morphogenesis and NO sensing
*DNA repair*					
740,026	*nfi*	590	−2.0	1.6E-10	Endonuclease V
*Amino acid transport and metabolism*					
10,028	*carB*	490	−2.3	3.8E-08	Carbamoyl-phosphate synthase, large subunit
180,040	*artJ*	878	−4.6	6.2E-25	Arginine transporter subunit; periplasmic-binding component of ABC superfamily
20,008	*leuC*	96	−2.1	0.0001	3-isopropylmalate dehydratase (isomerase), subunit with LeuD
270,021	*trpB*	302	−2.0	0.0003	Tryptophan synthase, beta subunit
270,022	*trpC*	244	−2.0	0.002	Fused indole-3-glycerolphosphate synthetase; N-(5-phosphoribosyl) anthranilate isomerase
270,023	*trpD*	222	−2.1	0.0003	Fused glutamine amidotransferase (component II) of anthranilate synthase; anthranilate phosphoribosyl transferase
270,024	*trpE*	173	−2.8	1.0E-06	Component I of anthranilate synthase
40,007	*metQ*	954	−2.1	1.9E-05	DL-methionine transporter subunit; periplasmic-binding component of ABC superfamily
40,009	*metN*	322	−2.8	8.1E-11	DL-methionine transporter subunit; ATP-binding component of ABC superfamily
430,005	*hisD*	257	−2.3	1.0E-09	Bifunctional histidinal dehydrogenase and histidinol dehydrogenase
430,006	*hisC*	59	−2.5	0.0004	Histidinol-phosphate aminotransferase
530,047	*argA*	172	−2.6	6.7E-07	Fused acetylglutamate kinase homolog (inactive); amino acid N-acetyltransferase
530,066	*lysA*	568	−2.1	4.9E-05	Diaminopimelate decarboxylase, PLP-binding
610,023	*argG*	754	−2.5	6.0E-09	Argininosuccinate synthetase
610,063	*gltD*	168	−2.0	6.2E-05	Glutamate synthase small chain
660,006	*metL*	2959	−3.0	0.0003	Fused asparto kinase II; homoserine dehydrogenase II
660,007	*metB*	251	−2.1	0.0003	Cystathionine gamma-synthase, PLP-dependent
700,021	*metE*	3087	−2.5	2.1E-06	5-methyl tetra hydro pteroyl tri glutamate-homocysteine S-methyltransferase
700,022	*metR*	275	−2.1	3.4E-05	DNA-binding transcriptional activator, homocysteine-binding
700,077	*ilvC*	611	−3.2	4.8E-11	Ketol-acid reductoisomerase, NAD(P)-binding
700,079	*ilvA*	444	−2.5	5.5E-09	Threonine deaminase
700,081	*ilvE*	402	−2.0	1.1E-05	Branched-chain amino-acid aminotransferase
700,083	*ilvB(G)*	273	−2.0	0.0002	Acetolactate synthase I, large subunit
700,084	*ilvL*	5169	−2.6	4.9E-17	ilvG operon leader peptide
710,012	*asnA*	3488	−2.1	1.1E-12	Asparagine synthetase A
730,015	*argC*	450	--3.2	1.9E-15	N-acetyl-gamma-glutamylphosphate reductase, NAD(P)-binding
730,016	*argB*	154	−2.6	2.6E-07	Acetylglutamate kinase
730,017	*argH*	1040	−3.5	2.1E-13	Argininosuccinate lyase
760,070	*adiC*	582	−3.7	2.1E-13	Arginine/agmatine antiporter
760,072	*adiA*	224	−2.6	3.1E-08	Biodegradative arginine decarboxylase
800,012	*argI*	112	−6.5	1.9E-20	Ornithine carbamoyltransferase 1
*Carbohydrate metabolism and energy production*					
470,020	*glpA*	363	−2.0	0.001	sn-glycerol-3-phosphate dehydrogenase (anaerobic), large subunit, FAD/NAD(P)-binding
710,165	*lldD*	973	−2.1	1.1E-12	L-lactate dehydrogenase, FMN linked
710,166	*lldR*	123	−2.5	6.5E-06	DNA-binding transcriptional repressor
710,167	*lldP*	430	−2.1	8.1E-06	L-lactate permease
*bacterial pathogenicity*					
230,055	*yccC*	1840	−2.1	9.6E-07	Cryptic autophosphorylating protein tyrosine kinase Etk
230,056	*etp*	214	−2.1	0.002	Phosphotyrosine-protein phosphatase
*Inorganic ion transport and metabolism*					
430,088	*rncA*	68	−2.0	0.003	Nickel and cobalt resistance
560,020	*fecR*	116	−3.0	1.7E-08	Transmembrane signal transducer for ferric citrate transport; KpLE2 phage-like element
620,109	*feoB*	997	−2.0	1.1E-05	Fused ferrous iron transporter, protein B: GTP-binding protein; membrane protein
620,179	*nikB*	148	−2.1	0.0004	Nickel transporter subunit; membrane component of ABC superfamily
620,180	*nikC*	113	−2.3	1.7E-05	Nickel transporter subunit; membrane component of ABC superfamily
620,181	*nikD*	180	−2.0	0.0006	Nickel transporter subunit; ATP-binding component of ABC superfamily
*Cell wall/membrane biogenesis*					
20,017	*ftsL*	485	−2.3	1.3E-07	Membrane bound cell division protein at septum containing leucine zipper motif
20,020	*murF*	436	−2.3	5.5E-08	UDP-N-acetylmuramoyl-tripeptide:D-alanyl-D-alanine ligase
*Transcription*					
180,070	*lrp*	2442	−3.0	1.7E-15	DNA-binding transcriptional dual regulator, leucine-binding

^a^FC is the fold change of the genes that exhibit significant (FC ≤ −2, false discovery rate (FDR) ≤ 0.005, minimum normalized read count = 10) differential expression. Only discussed genes in our study are shown on this table

^b^Adjusted *p*-value for multiple testing with the Benjamini-Hochberg procedure which controls FDR

**Table 2:** Genes significantly down-regulated in *Escherichia coli* O157:H7 EDL933 in samples with microbiota compared to those without microbiota

Locus (Z #)	Gene name	Mean of normalized counts	FC^a^	adj. *p* ^b^	Function or product
*Nitrate metabolism and nitrite detoxification*					
1107	*hcp*	270	−2.5	3.2E-08	Hybrid-cluster [4Fe-2S-2O] protein in anaerobic terminal reductases
*Amino acid transport and metabolism*					
0037	*carA*	369	−2.3	1.8E-07	Carbamoyl phosphate synthetase small subunit, glutamine amidotransferase
1090	*artJ*	1305	−4.0	8.4E-15	Arginine transporter subunit; periplasmic-binding component of ABC superfamily
1093	*artI*	407	−2.0	0.0002	Arginine transporter subunit; periplasmic-binding component of ABC superfamily
3181	*hisG*	221	−2.0	0.001	ATP phosphoribosyltransferase
3569	*hisM*	208	−2.0	0.0008	Histidine/lysine/arginine/ornithine transporter subunit; membrane component of ABC superfamily
4135	*argA*	737	−2.5	2.7E-07	Fused acetylglutamate kinase homolog (inactive); amino acid N-acetyltransferase
4534	*argG*	972	−3.0	9.9E-12	Argininosuccinate synthetase
5285	*ilvC*	773	−2.0	2.1E-05	Ketol-acid reductoisomerase. NAD(P)-binding
5515	*argE*	448	−2.5	2.8E-09	Acetylornithine deacetylase
5516	*argC*	1480	−3.7	1.4E-16	N-acetyl-gamma-glutamylphosphate reductase, NAD(P)-binding
5517	*argB*	533	−2.8	1.1E-09	Acetylglutamate kinase
5518	*argH*	1496	−2.5	7.2E-06	Argininosuccinate lyase
5719	*adiA*	539	−2.1	0.001	Biodegradative arginine decarboxylase
5866	*argI*	404	−2.5	0.0006	Ornithine carbamoyltransferase 1
*bacterial pathogenicity*					
3200	*peR*	739	−2.0	6.9E-05	Perosamine synthetase Per

^a^FC is the fold change of the genes that exhibit significant (FC ≤ −2, false discovery rate (FDR) ≤ 0.005, minimum normalized read count = 10) differential expression. Only genes discussed in our study are shown on this table

^b^Adjusted *p*-value for multiple testing with the Benjamini-Hochberg procedure which controls FDR

#### Genes significantly down-regulated in ground beef with microbiota

##### Genes involved in amino acid transport and biosynthesis

For O26_21765_, 31 of the 97 genes known, in *E. coli*, to encode the enzymes needed for amino acid biosynthesis [43] were down-regulated in ground meat with microbiota (Fig. 3; Table 1). For example, genes implicated in tryptophan biosynthesis (*trpE*, *trpD*, *trpC*, *trpB* and *trpA*), methionine biosynthesis, transport or regulation (*metBELQNR*), biosynthesis of glutamate (*gltD*), lysine (*lysA*), and arginine (*argABCIHG* and *carB*) and genes which encode the enzymes of the isoleucine and valine synthesis pathway (*ilvGEALG*) were down-regulated. For O157_EDL933_, eight genes implicated in the arginine biosynthesis pathway were down-regulated (*argABCEIHG* and *carA*) (Table 2). The *artI* gene encoding arginine transporter subunit and the *hisM* gene encoding histidine/lysine/arginine/ornithine transporter subunit were also down-regulated. The *hisCD* and *hisG* genes of the histidine biosynthetic pathway were down-regulated in strains O26_21765_ and O157_EDL933_ respectively. The *ilvC* and *artJ* genes, which encode Ketol-acid reductoisomerase and arginine transporter subunit respectively, were down-regulated in both strains. The *adiC* and *adiA* genes were also both down-regulated in O26_21765_. Arginine produced or imported into the cell by AdiC is decarboxylated inside the cell by AdiA to form agmatine, releasing CO_2_ and replacing it with a proton. The *leuC* and *asnA* genes, which encode 3-isopropylmalate dehydratase and asparagine synthetase A respectively, were also down-regulated in O26_21765_. The *lrp* gene encoding leucine-responsive regulatory protein (Lrp) which is a global regulator of *E. coli* metabolism was down-regulated in O26_21765_. The repression of this regulatory gene correlated well with the reduced transcript levels of its target genes, notably the decreased expression of several genes of the amino acid biosynthesis pathways (Table 1). Expression of the *lrp* gene is known to be regulated in part by the nutrients available to the cell, and is decreased in glucose minimal media enriched with amino acids [44]. The repression of genes involved in amino acid biosynthesis can be explained by the repression of *lrp* in ground meat with microbiota. The hypothesis proposed is that the hydrolyzed proteins produced by the activity of proteolytic enzymes and peptidases of the background microbiota may enrich the meat with peptides and amino acids and so inhibit the expression of *lrp*. These data are in accordance with a study led by Tao et al. (1999) which indicated that the expression of many genes involved in the biosynthesis of building blocks, most notably the amino acid biosynthesis pathways, is repressed in high-nutrient environments, as appears to be the case in the present study.

##### Genes involved in carbohydrate metabolism and energy production

Genes implicated in L-lactate metabolism were down-regulated in O26_21765_ (*lldR, lldP* and *lldD*). L-lactate dehydrogenase (encoded by *lldD*) is a peripheral membrane protein that catalyzes the oxidation of L-lactate to pyruvate. This protein and L-lactate permease (encoded by the *lldP* gene) allow *E. coli* to grow in a medium containing L-lactate as the sole carbon source [45]. Transcription of *glpA*, which encodes a sn-glycerol-3-phosphate (G3P) dehydrogenase, GlpA, was also repressed in O26_21765_ (Table 1). Under anaerobic conditions, G3P dehydrogenase GlpA converts G3P to dihydroxyacetone phosphate [46]. This latter compound is converted into glyceraldehyde-3P entering the gluconeogenesis pathway [47]. Thus, in ground meat, the presence of microbiota seems to repress the transcription of several genes involved in the assimilation of gluconeogenic substrates such as glycerol-3P and L-lactate. These metabolites could be consumed by the background microbiota.

##### Genes implicated in cell wall/membrane biogenesis

The *murF* gene encoding the MurF amide ligase enzyme was repressed in O26_21765_ (Table 1). MurF has been shown to be required for catalyzing the final step in the synthesis of bacterial cell wall peptidoglycan and to be essential for bacterial survival [48–51]. The *ftsL* gene encoding the conserved division protein FtsL was also down-regulated in O26_21765_ (Table 1). FtsL, rather than serving simply as a protein scaffold within the divisome along with its partners FtsB and FtsQ, functions as part of a sensing mechanism that promotes the onset of cell wall remodeling processes needed for the initiation of cell constriction once assembly of the divisome complex is deemed complete [52–54].

In the present study we demonstrated that *murF* and *ftsL* genes were down-regulated in strain O26_21765_ in meat with microbiota. The microbiota may exert an inhibitory action on EHEC strains by interfering with the cytokinetic ring apparatus and the synthesis of bacterial cell wall peptidoglycan. Many studies have shown that the growth of EHEC strains in ground beef may be inhibited by background microbiota initially present at levels greater than that of the pathogen *E. coli* [55, 56]*.* In our study, the low level of natural microbiota compared to strain O26_21765_ might not sufficiently affect the expression of *murF* and *ftsL* genes to halt growth. Both strains (O157_EDL933_ and O26_21765_) were added at an initial level of about 3 logs above that of the natural microbiota. After 7 days of incubation at 12 °C, about 8 log CFU/g of each type of natural microbiota (aerobic and LAB) were counted whereas 9 log CFU/g of each EHEC strains were enumerated. In the tested conditions, the presence of the meat microbiota at an initial level of about 10^4^ CFU/g did not impair the growth of either O157_EDL933_ or O26_21765_ (See Additional file 1: Table S1; Additional file 2: Table S2).

##### Genes involved in nitrate metabolism and nitrite detoxification

The mean raw nitrate content of fresh meat ranged from 18.7 to 38.5 mg/kg [57]. Microorganisms can reduce nitrate to nitrite, which is partially oxidized to nitrate by sequestering oxygen [58]. The nitrite undergoes chemical reactions that lead to reactive nitrogen species (RNS), including NO [58, 59]. These RNSs can interact with and damage numerous targets, including tyrosine residues, thiols and nucleotide bases [60]. Two down-regulated genes, *hcr* and *hcp*, were identified in strains O26_21765_ and O157_EDL933_ respectively (Tables 1 and 2). They encode the hybrid cluster protein (HCP) and its NADH oxidoreductase (HCR), respectively, which are supposed to be involved in nitrite or nitrate reduction by an as yet unidentified reaction [61]. In *E. coli*, *hcp* has been found to be induced in presence of nitric oxide (NO) under both aerobic and anaerobic conditions [62]. Because of the cytotoxic effects of NO and its derivatives, *E. coli* has two prominent ways to detoxify NO: a nitric-oxide reductase (NorVW) and the flavohemoglobin Hmp. The *norVW* genes encode flavorubredoxin and flavorubredoxin reductase, respectively, which convert NO to nitrous oxide. In O157_EDL933_, the *norV* gene has a 154 bp deletion, resulting in a frameshift mutation. In O26_21765_, the *norR* gene, encoding an NO-responsive regulator (NorR) [63, 64], was down-regulated (Table 1).

In our study the *ytfE* gene was down-regulated in O26_21765_. The *ytfE* gene is predicted to encode for a cytoplasmic protein, and is annotated as a regulator of cell morphology and NO sensing [16, 65]. Those authors also showed that YtfE is of major importance in *E. coli*’s response to NO [65].

Other genes that have shown up-regulation in response to nitrosative stress [62, 66] were down-regulated in O26_21765_. These were the four *metBELR* genes, involved in methionine biosynthesis or regulation; the *gltD* gene, which encodes glutamate synthase (NADPH) small chain precursor; *grxA*, encoding glutaredoxin 1 redox coenzyme ribonucleotide reductase; and *ybdL*, encoding a PLP-dependent methionine aminotransferase (Table 1).

Expression of the *nfi* gene, encoding endonuclease V (EndoV), was also repressed in O26_21765_ grown in ground beef with microbiota. EndoV is a ubiquitous protein responsible for the specific cleavage at the second phosphodiester bond 3′ to inosine [67, 68]. In *E. coli*, many studies have shown that EndoV prevents mutations from nitrosative deamination during nitrate/nitrite respiration [69].

In ground beef with microbiota, raw nitrate content could be metabolized by background microbiota in the meat samples prior to their contamination with EHECs. The up-regulated NO response mechanisms in ground beef without microbiota may allow the pathogen to adapt to sublethal environmental conditions and enhance its resistance to chemicals typically used as preservatives.

##### Genes implicated in the bacterial pathogenicity of EHECs

The *peR* gene was down-regulated in strain O157_EDL933_ (Table 2). This gene encodes a perosamine synthetase essential for the production of O antigen. As described by Bilge et al., (1996), an O157:H7∆*peR* mutant is significantly more adherent to HeLa cells than is its *E. coli* O157:H7 parent [70], the mutant strain being deficient in the O antigen. The O side chains of bacterial lipopolysaccharide (LPS) may physically hinder contact between the outer membrane and eukaryotic cells. Because of the conserved nature of most EHEC O157 strains, such factors might explain the higher prevalence of O157 EHECs in bovine meat (Zoonose EFSA, 2014). Nevertheless, more data are needed on other O157 EHEC strains.

With O26_21765_, the *etk* and *etp* genes, which encode a protein-tyrosine kinase, Etk, and a phosphotyrosine-protein phosphatase, Etp, respectively, were down-regulated (Table 1). Various studies have shown that pathogenicity seems to be positively correlated with the level of tyrosine phosphorylation [71, 72]. Increasing evidence supports the idea that tyrosine phosphorylation contributes to several key steps in the infection process, such as adhesion to the host and regulation of pathogenic functions [73–75].

In our study we demonstrated that the expression of the O side chains of bacterial LPS in O157_EDL933_, and the level of tyrosine phosphorylation in O26_21765_ were down-regulated in meat with microbiota. Various studies have shown that, the O side chains of bacterial LPS and the level of tyrosine phosphorylation are correlated with the capacity of bacteria to adhere to the eukaryotic cells. Based on these studies, our hypothesis supports the idea that strain O26_21765_ might adhere less well to beef tissue with microbiota than O157_EDL933_. These data are in accordance with the higher prevalence of O157 compared to O26 EHECs in ground beef.

##### Genes implicated in inorganic ion transport and metabolism

Iron, nickel and other inorganic ions have various roles in cellular biochemistry. In particular, they are well known as co-factors with a key role in bacterial virulence. For example, it has been established that iron acquisition genes are important contributors to the virulence of uropathogenic *E. coli* (UPECs) [76]. In our study, several relevant genes were down-regulated in O26_21765_: the *nikBCD* genes, encoding one part of the *E. coli* NikABCDE permease. This permease is known to be maximally expressed under anaerobic condition when intracellular nickel is scarce [77, 78]; the *rcnA* gene, encoding a membrane bound polypeptide, RcnA, described as a cobalt and nickel efflux system in *E. coli*, [79]; the *feoB* gene, encoding the polytopic membrane protein, FeoB, which is essential for Fe(II) uptake [80] (note that *feoB* has been described as one of the most prevalent virulence genes in UPECs isolated from patients with community-acquired urinary tract infection [81]); and the *fecR* gene, encoding the FecR protein, the sensor that recognizes iron (III) dicitrate in the periplasm (Table 1) [82].

The results of the transcriptomic analysis of O26_21765_ suggest that iron and nickel could be more bioavailable in ground beef with microbiota and their transporters might be repressed in response to these external stimuli.

##### Other down-regulated genes

Three (ECO26H_v1_590016; 690,023 and 710,001) and two (Z4363 and 5408) genes annotated as putative transcriptional regulators were down-regulated respectively in O26_21765_ and O157_EDL933_ strains (Fig. 3; Fig. 4; Additional file 5: Table S5 and Additional file 7: Table S7). Among them, one (*yihL*) is shared by the two strains. Several other genes (ECO26H_v1_110033; 200,020; 370,007; 430,155; 710,101 and 750,064 in O26_21765_; Z1966 and 3199 in O157_EDL933_) were also down-regulated (Additional file 5: Table S5 and Additional file 7: Table S7). Their probable effects as a response to an environmental signal were not discussed in the present study because their functional role has not yet been fully characterized. Moreover, their annotation is based on the presence of conserved amino acid motifs and structural features or limited homology.

Eleven other genes (ECO26H_v1_120023; 210,002; 220,017; 340,120; 430,059; 500,297; 580,076; 60,012; 670,016; 80,078 and p30071), annotated as hypothetical or conserved proteins of unknown function, were down-regulated in the O26_21765_ strain (Additional file 5: Table S5). Only one (ECO26H_v1_ p30071) did not show any significant sequence identity with any of the known genes in the O157_EDL933_ strain.

#### Genes significantly up-regulated in ground meat with microbiota

Preamble. This is the first study to use strand-specific RNA-seq to investigate the effects of ground beef background microbiota on the overall gene expression of EHEC strains O26_21765_ and O157_EDL933_. To reduce the effect of spurious alignment and crosstalk between the reference genome of EHEC and other similar genomes, the genes that were transcriptionally up-regulated in the presence of microbiota were filtered for *E. coli* specific genes (Fig. 1). The down-regulated genes we identified could not be affected by spurious alignment or crosstalk between the reference genomes and other similar genomes, so it was not necessary to apply this filter. A spurious alignment could not generate a false down-regulated gene.

We therefore discarded from our study genes that we expected to be up-regulated in EHEC strains owing to the possibility of spurious alignment and crosstalk between the reference genomes and other similar genomes identified at the genus level by 16S metagenomics analysis. The criterion whether or not to discard an up-regulated gene was a score of nucleotide identity (between the altered gene and any other gene provided by GenBank as being part of the genome sequencing data of the identified genus by 16S meta-genomic analysis) of more than 50% across 80% or more of their length.

This constraint also influenced our sequencing strategy, sequencing mode (paired*-*end sequencing), library-type (strand-specific cDNA library) and choice of the read length (125 bp). The paired-end strand-specific RNA-Seq with a read length of 125 bp allows for higher levels of specific alignments, thus reducing the possibility of spurious alignment between the reference genome and other similar genomes. Reads with more than one reported alignment were not counted for any feature. We also discarded up-regulated genes with identity to any other gene identified as being part of genome sequencing data provided by GenBank and corresponding to the most abundant bacteria identified by 16S metagenomics analysis (Figs. 3 and 4). We discarded genes with more than 50% nucleotide identity across 80% or more of their length (For discarded genes see Additional file 6: Table S6 and Additional file 8: Table S8). Of the 28 and 35 genes induced in strains O26_21765_ and O157_EDL933_ respectively, 14 and 12 passed the filter and are discussed in this study (Tables 3 and 4).

**Table 3:** Genes significantly up-regulated in *Escherichia coli* O26:H11 21,765 in samples with microbiota compared to those without microbiota

Locus (ECO26H_v1_ #)	Gene name	Mean of normalized counts	FC^a^	adj. *p* ^b^	Function or product
*Unknown or hypothetical proteins*					
260022	*ymgB*	101	2.1	0.0001	Hypothetical protein
310027	*ydfP*	34	2.5	0.003	Conserved hypothetical protein; Qin prophage
340082	*ydhS*	506	2.0	0.0002	Conserved hypothetical protein; putative FAD/NAD(P)-binding domain
400008	*yecT*	105	2.1	0.0002	Hypothetical protein
420013	*yeeE*	55	2.3	0.001	Conserved hypothetical protein; putative inner membrane protein
50014	*_*	102	2.1	0.002	Conserved protein of unknown function
510037	*_*	162	2.5	3.6E-06	Protein of unknown function
590009	*_*	61	2.1	0.004	Conserved protein of unknown function
p40014	*_*	591	2.0	0.0002	Conserved protein of unknown function
*Other up-regulated genes*					
250005	_	48	2.3	0.001	T3SS secreted effector NleG-like protein
270179	*ydcX*	105	2.3	8.8E-06	Putative inner membrane protein
500243	*yfhR*	250	2.0	7.1E-05	Putative peptidase
50040	*yhhI*	106	2.1	0.0007	Putative transposase
p40035	*_*	99	2.3	0.0002	Pilus assembly protein

^a^FC is the fold change of the genes that exhibit significant (FC ≥ 2, false discovery rate (FDR) ≤ 0.005, minimum normalized read count = 10) differential expression. Only genes passed the filter are shown on this table. Expected genes to be differentially expressed due to the possibility of spurious alignment, and crosstalk between the reference genome and other similar genomes identified at genus level by 16S meta-genomic analysis, were discarded from this study (For discarded genes see Additional file 6: Table S6)

^b^Adjusted *p*-value for multiple testing with the Benjamini-Hochberg procedure which controls FDR

**Table 4:** Genes significantly up-regulated in *E. coli* O157:H7 EDL933 in samples with microbiota compared to those without microbiota

Locus (Z #)	Gene name	Mean of normalized counts	FC^a^	adj. *p* ^b^	Function or product
*Antibiotic resistance*					
2169	*marB*	78	2.6	8.2E-06	Conserved hypothetical protein associated with multiple antibiotic resistance operon
2170	*marA*	133	2.1	2.1E-05	DNA-binding transcriptional dual activator of multiple antibiotic resistance
*Response to stress*					
2478	*pspD*	74	2.6	2.2E-05	Peripheral inner membrane phage-shock protein
*Hypothetical proteins*					
1402	*ymcB*	102	2.1	0.0004	Conserved hypothetical protein
2695	*ydhS*	1115	2.0	0.0001	Conserved hypothetical protein; putative FAD/NAD(P)-binding domain
2774	*ydjR*	206	2.3	0.0004	Conserved hypothetical protein
3175	*yeeE*	143	2.8	1.7E-07	Conserved hypothetical protein; putative inner membrane protein
5712	*yjcZ*	173	2.1	0.0002	Conserved hypothetical protein
*Other up-regulated genes*					
0043	*caiC*	204	2.0	0.0005	Putative crotonobetaine CoA ligase:carnitine CoA ligase
1987	*ldrB*	176	2.0	0.001	Fragment of small toxic polypeptide (partial)
3266	*yegX*	100	2.3	0.0002	Putative membrane-bound hydrolase
3660	*yfeA*	3551	2.0	2.9E-05	Putative diguanylate cyclase

^a^FC is the fold change of the genes that exhibit significant (FC ≥ 2, false discovery rate (FDR) ≤ 0.005, minimum normalized read count = 10) differential expression. Only genes passed the filter are shown on this table. Expected genes to be differentially expressed due to the possibility of spurious alignment, and crosstalk between the reference genome and other similar genomes identified at genus level by 16S meta-genomic analysis, were discarded from this study (For discarded genes see Additional file 8: Table S8)

^b^Adjusted *p*-value for multiple testing with the Benjamini-Hochberg procedure which controls FDR

##### Genes implicated in a detoxification role in EHECs

For O157_EDL933_, the *marA* and *marB* genes, which encode a DNA-binding transcriptional dual activator of multiple antibiotic resistance and a conserved hypothetical protein associated with a multiple antibiotic resistance operon, respectively, were up-regulated (Table 4). The expression of these genes as part of the antibiotic resistance machinery could be activated in response to external stimuli [83], presence of an antibiotic at a sub-lethal concentration being the most common stimulus. In our 16S metagenomics study, the genus *Serratia* represented about 1.5% of the bacterial population in the ground beef sample inoculated with O157_EDL933_ (Fig. 2; Additional file 3: Table S3). Most *Serratia* spp. have been shown to produce antibiotics. For example, production of carbapenem antibiotics has been demonstrated in *Serratia* sp. ATCC 39006 [84]. Production of antibiotics has also been reported in *Serratia plymuthica* RVH1 [85] and *Serratia marcescens* strain 12 [86]. These observations suggest that the presence of ground beef background microbiota might induce multidrug resistance in EHECs by stimulating transcription of *E. coli* drug resistance genes such as *marA*, which has been described as sufficient to give *E. coli* multiple antibiotic resistance [87].

##### Genes implicated in stress response

The *pspD* gene encoding the peripheral inner membrane phage-shock protein pspD was up-regulated in O157_EDL933_ (Table 4). Expression of the *pspD* gene is known to be associated with membrane stress and in response to conditions such as phage attack, heat shock and hyperosmotic stress, and exposure to certain metabolites [88, 89]. Thus, the presence of adventitious microbial activity or phage could induce specific stresses in *E. coli*. However, this needs further investigation.

##### Other up-regulated genes

Several other genes were up-regulated (ECO26H_v1_250,005; p40035; 500,243; 50,040 and Z0043; 3266 respectively, in the O26_21765_ and O157_EDL933_ strains), but their functional roles, based on the presence of conserved amino acid motifs, structural features or limited identity, have not yet been fully characterized (Tables 3 and 4). The effect of their over-expression in response to an environmental signal needs to be identified and they are now new candidates for detailed functional description.

##### Genes encoding hypothetical proteins

Of the 14 and 12 genes studied, 9 and 5 were annotated as unknown or hypothetical proteins in O26_21765_ and O157_EDL933_ strains, respectively (Tables 3 and 4). Two genes (*yeeE* and *ydhS*) were shared between the two strains. Six other (ECO26H_v1_260,022; 310,027; 50,014; 510,037; 590,009 and p40014) identified in O26_21765_ strain didn’t show any significant sequence identity with any gene of the other one. In EHEC, about one third of the genes are still annotated as hypothetical [89]. Genes annotated as hypothetical and up-regulated in presence of naturel microbiota, are now new candidates for a detailed functional description.

## Conclusions

This article demonstrates how RNA-Seq coupled with 16S metagenomics analysis can be used to discover new and relevant impacts on the functions of an individual microbe within a complex environmental community. The methodology developed was designed for describing the effects of ground beef background microbiota on the overall gene expression of EHEC strains O26_21765_ and O157_EDL933_ by comparison with their behavior in beef with no microbiota. For our experimental design, the choice of two strains, one isolated from Michigan ground beef (O157_EDL933_) and one from human fecal samples (O26_21765_), enabled us to gain information of continuing importance for understanding the biology of EHECs in general and the strain-specific adaptations of strains O157_EDL933_ and O26_21765_ in particular.

In strain O26_21765_, 95 genes were expressed at significantly lower levels in ground beef samples with microbiota, and 28 were significantly up-regulated. The corresponding numbers for down- and up-regulated genes in O157_EDL933_ were 21 and 25. Together, all the genes identified in the present study serve as a starting point for developing testable hypotheses about the mechanisms underlying the organism’s adaptations to different habitats, including the evolution of its virulence. Moreover, genes that are altered in presence of natural microbiota, notably genes for the category of the hypothetical proteins, are now new targets for studying interactions between microorganisms. Finally, *murF* and *ftsL* exemplarily show that transcriptome profiling will be a powerful technique for designing novel therapeutic approaches, since treating an EHEC infection with antibiotics can potentiate the disease.

Tremendous diversity exists within the meat microbiota and changing experimental conditions such as storage conditions will alter the microbial population [90, 91] and so change its interaction mechanisms. As this new methodology is not tied to particular conditions, it can be used with any other conditions, where it would screen for new interaction mechanisms.

## Additional files

Additional file 1: Table S1. Total viable counts of natural microbiota detected in the ground beef held at 12 °C and prepared from the outer part of the muscle. (DOC 29 kb)

Additional file 2: Table S2. Population over time of two strains of enterohemorrhagic *Escherichia coli* (O157:H7 EDL933 and O26:H11 21,765) grown in ground beef held at 12 °C and prepared from the outer or inner part of the muscle. (DOC 30 kb)

Additional file 3: Table S3. Genus level distribution of sequences based on the 16S rRNA gene libraries constructed from a ground beef sample with natural microflora (A) and ground beef samples inoculated with *E. coli* O26:H11 21,765 (B) or *E. coli* O157:H7 EDL933 (C) strains. (DOC 55 kb)

Additional file 4: Table S4. Summary of enterohemorrhagic *Escherichia coli* cDNA samples sequenced in ground beef with or without microbiota (DOC 41 kb)

Additional file 5: Table S5. Others down-regulated genes in *Escherichia coli* O26:H11 21,765 in samples with microbiota compared to those without microbiota. (DOC 95 kb)

Additional file 6: Table S6. Discarded up-regulated genes in *Escherichia coli* O26:H11 21,765 in samples with microbiota compared to those without microbiota. (DOC 51 kb)

Additional file 7: Table S7. Others down-regulated genes in *Escherichia coli* O157:H7 EDL933 in samples with microbiota compared to those without microbiota. (DOC 36 kb)

Additional file 8: Table S8. Discarded up-regulated genes in *Escherichia coli* O157:H7 EDL933 in samples with microbiota compared to those without microbiota. (DOC 62 kb)

## Abbreviations

BHI: Brain heart infusion
bp: Base pairs
BPW: Buffered peptone
cDNA: Complementary DNA
CDS: Coding DNA sequence
CFU: Colony-forming units
DE: Differential expression
DNA: Deoxyribonucleic acid
*E.*: *coli*
EFSA: European food safety authority
EHEC: Enterohemorrhagic *Escherichia coli*
EU: European Union
FDR: False discovery rate
GLM: Generalized linear model
HCP: Hybrid cluster protein
HUS: Hemolytic-uremic syndrome
LAB: Lactic acid bacteria
LPS: LipoPolySaccharide
NO: Nitric oxide
ORF: Open reading frame
OUT: Operational taxonomic unit
PCA: Plate-count agar
PCR: Polymerase chain reaction
RNS: Reactive nitrogen species
rRNA: Ribosomal ribonucleic acid
RTE: Ready-To-Eat
STEC: Shiga toxine *E. coli*
UPEC: UroPathogenic
