# Supplementary material for: Strand-specific transcriptomes of Enterohemorrhagic Escherichia coli in response to interactions with ground beef microbiota: interactions between microorganisms in raw meat
Source: BMC Genomics. 2017 Aug 3;18:574. doi: 10.1186/s12864-017-3957-2 (PMC5543532; doi:10.1186/s12864-017-3957-2)
Supplement: Supplementary file 1 — Total viable counts of natural microbiota detected in the ground beef held at 12 °C and prepared from the outer part of the muscle. (DOC 29 kb) [file 12864_2017_3957_MOESM1_ESM.doc]

Table S1: Total viable counts of natural microbiota detected in the ground beef held at 12°C and prepared from the outer part of the muscle

| Sampling time (Day/inoculation) | Aerobic bacteria  (log CFU/g) | LAB  (log CFU/g) |
| --- | --- | --- |
| 1* | 4.14 ± 0.04 | 3.88 ± 0.04 |
| 7 | 8.30 ± 0.43^1^  8.35 ± 0.49^2^ | 8.25 ± 0.15^1^  8.51 ± 0.10^2^ |

*Sample obtained 15 min before inoculation with enterohemorrhagic *Escherichia coli* (EHEC) strains.

^1^ and ^2^ Enumeration of natural microflora in ground meat inoculated respectively by EHEC O26:H11 21765 and O157:H7 EDL933 strains.

All counts were performed in duplicate.
