# Supplementary material for: Strand-specific transcriptomes of Enterohemorrhagic Escherichia coli in response to interactions with ground beef microbiota: interactions between microorganisms in raw meat
Source: BMC Genomics. 2017 Aug 3;18:574. doi: 10.1186/s12864-017-3957-2 (PMC5543532; doi:10.1186/s12864-017-3957-2)
Supplement: Supplementary file 2 — Population over time of two strains of enterohemorrhagic Escherichia coli (O157:H7 EDL933 and O26:H11 21,765) grown in ground beef held at 12 °C and prepared from the outer or inner part of the muscle. (DOC 30 kb) [file 12864_2017_3957_MOESM2_ESM.doc]

Table S2: Population over time of two strains of enterohemorrhagic *Escherichia coli* (O157:H7 EDL933 and O26:H11 21765) grown in ground beef held at 12°C and prepared from the outer or inner part of the muscle

| Sampling time  (Day/inoculation) | Origin of the sample  /muscle | O26:H11 21765  (log CFU/g) | O157:H7 EDL933 (log CFU/g) |
| --- | --- | --- | --- |
| 1* | Outer or inner part | 7.56 ± 0.17 | 7.31 ± 0.43 |
| 7 | Outer part | 9.68 ± 0.01 | 9.55 ± 0.32 |
|  | Inner part | 8.38 ± 0.09 | 9.52 ± 0.07 |

*Sample obtained 15 min after inoculation with enterohemorrhagic *Escherichia coli* (EHEC) strains.

All counts were performed in duplicate.
