# Supplementary material for: Strand-specific transcriptomes of Enterohemorrhagic Escherichia coli in response to interactions with ground beef microbiota: interactions between microorganisms in raw meat
Source: BMC Genomics. 2017 Aug 3;18:574. doi: 10.1186/s12864-017-3957-2 (PMC5543532; doi:10.1186/s12864-017-3957-2)
Supplement: Supplementary file 3 — Genus level distribution of sequences based on the 16S rRNA gene libraries constructed from a ground beef sample with natural microflora (A) and ground beef samples inoculated with E. coli O26:H11 21,765 (B) or E. coli O157:H7 EDL933 (C) strains. (DOC 55 kb) [file 12864_2017_3957_MOESM3_ESM.doc]

Table S3 : Genus level distribution of sequences based on the 16S rRNA gene libraries constructed from a ground beef sample with natural microflora (A) and ground beef samples inoculated with *E. coli* O26:H11 21765 (B) or *E. coli* O157:H7 EDL933 (C) strains

| Genus | A | B | C |
| --- | --- | --- | --- |
| *Escherichia* | 0.014 | 74.914 | 76.399 |
| *Serratia* | 79.355 | 1.330 | 3.170 |
| *Carnobacterium* | 8.796 | 8.801 | 9.838 |
| Enteric_Bacteria_cluster | 6.821 | 3.110 | 7.263 |
| *Kurthia* | 2.265 | 5.776 | 0.149 |
| *Lactococcus* | 0.404 | 4.448 | 1.699 |
| *Brochothrix* | 0.732 | 0.074 | 0.326 |
| *Unclassified* | 0.620 | 0.905 | 0.502 |
| *Marinilactibacillus* | 0.026 | 0.049 | 0.050 |
| *Enterobacter* | 0.098 | 0.120 | 0.192 |
| *Pectobacterium* | 0.093 | 0.001 | 0.003 |
| *Cronobacter* | 0.003 | 0.017 | 0.052 |
| *Klebsiella* | 0.011 | 0.009 | 0.007 |
| *Citrobacter* | 0.003 | 0.213 | 0.001 |
| *Raoultella* | 0.015 | 0.002 | 0.001 |
| *Vagococcus* | 0.013 | 0.008 | 0.011 |
| *Buttiauxella* | 0.598 | 0.001 | 0.001 |
| *Acinetobacter* | 0.003 | 0.022 | 0.003 |
| *Enterococcus* | 0.013 | 0.011 | 0.004 |
| *Azomonas* | 0.001 | 0.006 | 0.003 |
| *Pseudomonas* | 0.052 | 0.151 | 0.282 |
| *Paralactobacillus* | 0.001 | 0.007 | 0.001 |
| *Isobaculum* | 0.002 | 0.011 | 0.001 |
| *Yersinia* | 0.005 | 0.001 | 0.001 |
| *Desemzia* | 0.001 | 0.003 | 0.002 |
| *Macrococcus* | 0.053 | 0.001 | 0.001 |
| *Streptococcus* | 0.001 | 0.005 | 0.031 |
| *Leuconostoc* | 0.002 | 0.002 | 0.006 |
| *Melissococcus* | 0.001 | 0.002 | 0.001 |

All the sequences were classified at bootstrap value cutoff = 0.6.
