# Supplementary material for: Strand-specific transcriptomes of Enterohemorrhagic Escherichia coli in response to interactions with ground beef microbiota: interactions between microorganisms in raw meat
Source: BMC Genomics. 2017 Aug 3;18:574. doi: 10.1186/s12864-017-3957-2 (PMC5543532; doi:10.1186/s12864-017-3957-2)
Supplement: Supplementary file 4 — Summary of enterohemorrhagic Escherichia coli cDNA samples sequenced in ground beef with or without microbiota (DOC 41 kb) [file 12864_2017_3957_MOESM4_ESM.doc]

Table S4: Summary of enterohemorrhagic *Escherichia coli* cDNA samples sequenced in ground beef with or without microbiota

| Sequenced sample* | No. of total high quality reads | rRNA reads  (% of total high quality reads) | | No. of total mapped reads | | | No. of mapped  reads (aligned concordantly exactly 1 time) |
| --- | --- | --- | --- | --- | --- | --- | --- |
|  |  | Eukaryote | Bacteria | Genome | Plasmid | Total |  |
| O157:H7 F R1 | 26840438 | 78.8 | 1.2 | 3580787 | 40639 | 3621426 | 3376238 |
| O157:H7 F R2 | 19133205 | 77.5 | 1.0 | 2546362 | 36561 | 2582923 | 2423159 |
| O157:H7 F R3 | 22959300 | 80.4 | 1.0 | 2444413 | 41611 | 2486024 | 2327134 |
| O157:H7 S R1 | 28672177 | 59.1 | 0.9 | 8284056 | 114648 | 8290038 | 7796854 |
| O157:H7 S R2 | 28625308 | 67.0 | 0.6 | 5927130 | 81765 | 6008895 | 5709792 |
| O157:H7 S R3 | 24590542 | 70.3 | 0.9 | 4210967 | 65138 | 4276105 | 4040502 |
| O26:H11 F R1 | 57585347 | 80.9 | 1.0 | 4384280 | 286540 | 4670820 | 4415142 |
| O26:H11 F R2 | 63934898 | 80.5 | 1.4 | 4855786 | 305589 | 5161375 | 4850660 |
| O26:H11 F R3 | 63543012 | 81.6 | 0.9 | 4757568 | 294932 | 5052500 | 4761820 |
| O26:H11 S R1 | 22374029 | 77.2 | 2.3 | 1782257 | 109052 | 1891309 | 1782305 |
| O26:H11 S R2 | 17151954 | 74.5 | 1.8 | 1849699 | 110411 | 1960110 | 1843509 |
| O26:H11 S R3 | 59457919 | 78.1 | 1.3 | 5109706 | 303161 | 5412867 | 5094114 |

*O157:H7 and O26:H11 corresponding respectively to *E. coli* O157:H7 EDL933 and O26:H11 21765 strains; F, S corresponding respectively to ground beef with or without microbiota; R1, R2 or R3 corresponding to replicate number.
