# Supplementary material for: Strand-specific transcriptomes of Enterohemorrhagic Escherichia coli in response to interactions with ground beef microbiota: interactions between microorganisms in raw meat
Source: BMC Genomics. 2017 Aug 3;18:574. doi: 10.1186/s12864-017-3957-2 (PMC5543532; doi:10.1186/s12864-017-3957-2)
Supplement: Supplementary file 5 — Others down-regulated genes in Escherichia coli O26:H11 21,765 in samples with microbiota compared to those without microbiota. (DOC 95 kb) [file 12864_2017_3957_MOESM5_ESM.doc]

Table S5: Others down-regulated genes in *Escherichia coli* O26:H11 21765 in samples with microbiota compared to those without microbiota

| Locus  (ECO26H_v1_ #) | Gene name | Mean of normalized counts | FC^a^ | adj. *p*^b^ | Function or product |
| --- | --- | --- | --- | --- | --- |
| *Carbohydrate metabolism and energy production* | | | | | |
| 120032 | *sdhA* | 1798 | -2.3 | 3.7E-08 | Succinate dehydrogenase, flavoprotein subunit |
| 120036 | *sucC* | 1908 | -2.0 | 1.4E-06 | Succinyl-CoA synthetase beta chain |
| 30105 | *frdB* | 1528 | -2.5 | 8.7E-07 | Fumarate reductase (anaerobic), Fe-S subunit |
| 470051 | *nuoM* | 560 | -2.0 | 6.6E-07 | NADH dehydrogenase I chain M, membrane subunit |
| 470054 | *nuoJ* | 274 | -2.0 | 0.0001 | NADH:ubiquinone oxidoreductase, membrane subunit J |
| 470055 | *nuoI* | 336 | -2.0 | 9.1E-07 | NADH dehydrogenase I chain I, 2Fe-2S ferredoxin-relate |
| 470057 | *nuoG* | 1732 | -2.0 | 1.1E-06 | NADH:ubiquinone oxidoreductase, chain G |
| 470059 | *nuoE* | 292 | -2.0 | 1.5E-05 | NADH:ubiquinone oxidoreductase, chain E |
| 710093 | *uhpT* | 50 | -2.3 | 0.0003 | Hexose phosphate transporter |
| 790038 | *treB* | 91 | -2.0 | 0.0005 | Fused trehalose(maltose)-specific PTS enzyme: IIB component ; IIC component |
| 80061 | *cyoE* | 2400 | -2.3 | 2.4E-14 | Protoheme IX farnesyltransferase |
| *Other up-regulated genes* | | | | | |
| 110033 | *ybdH* | 302 | -2.3 | 2.1E-06 | Putative oxidoreductase |
| 120023 | *abrB* | 239 | -2.5 | 2.5E-09 | Putative membrane protein |
| 200020 | *_* | 57 | -2.1 | 0.001 | Putative dehydratase |
| 210002 | *_* | 366 | -2.3 | 1.5E-05 | Predicted protein (fragment) |
| 220017 | *_* | 370 | -2.3 | 5.5E-08 | Conserved protein of unknown function |
| 30061 | *cdaR* | 291 | -2.0 | 0.0002 | Fragment of DNA-binding transcriptional activator (part 1) |
| 340120 | *ydiE* | 57 | -2.6 | 0.0001 | Conserved hypothetical protein; putative hemin uptake protein |
| 370007 | *yeaU* | 98 | -2.1 | 0.0005 | Putative tartrate dehydrogenase |
| 430059 | *_* | 652 | -2.3 | 2.5E-08 | Conserved exported protein of unknown function |
| 430155 | *yeiQ* | 768 | -2.1 | 5.6E-06 | Putative sugar dehydrogenase, NAD-dependent |
| 500297 | *yfiM* | 768 | -2.6 | 3.1E-08 | Hypothetical protein |
| 580076 | *_* | 360 | -2.6 | 6.0E-09 | Predicted protein (fragment) |
| 590016 | *yqjI* | 410 | -2.1 | 6.6E-07 | Putative transcriptional regulator |
| 60012 | *_* | 1671 | -2.0 | 2.5E-07 | Conserved protein of unknown function |
| 670016 | *yiiS* | 1018 | -2.3 | 9.6E-07 | Conserved hypothetical protein |
| 690023 | *yihL* | 102 | -2.0 | 0.0006 | Putative DNA-binding transcriptional regulator |
| 700009 | *ubiD* | 2615 | -2.1 | 4.9E-06 | 3-octaprenyl-4-hydroxy benzoate decarboxylase |
| 710001 | *yieP* | 1277 | -2.0 | 1.4E-06 | Putative transcriptional regulator |
| 710101 | *setC* | 35 | -2.3 | 0.004 | Putative sugar efflux system |
| 750064 | *_* | 2683 | -2.1 | 5.7E-06 | Phage regulatory, Rha family protein |
| 780015 | *_* | 1273 | -2.0 | 1.5E-07 | Transposase (fragment) |
| 80078 | *ybaE* | 93 | -2.3 | 0.0005 | Conserved hypothetical protein |
| p30071 | *_* | 769 | -2.0 | 3.6E-05 | Conserved protein of unknown function |
| 520065 | *hycE* | 80 | -3.2 | 1.6E-05 | Hydrogenase 3, large subunit |
| 520066 | *hycD* | 27 | -3.0 | 0.001 | Hydrogenase 3, membrane subunit |
| 520067 | *hycC* | 73 | -2.3 | 0.001 | Hydrogenase 3, membrane subunit |
| 520068 | *hycB* | 16 | -4.6 | 9.1E-06 | Hydrogenase 3, Fe-S subunit |
| 520071 | *hypB* | 284 | -2.3 | 1.4E-06 | GTP hydrolase involved in nickel liganding into hydrogenases |
| 580003 | *hybF* | 256 | -2.0 | 0.0002 | Protein involved with the maturation of hydrogenases 1 and 2 |
| 760039 | *fdhF* | 344 | -2.8 | 3.0E-13 | Formate dehydrogenase-H, selenopolypeptide subunit |
| *Inorganic ion transport and metabolism* | | | | | |
| 430204 | *ccmA* | 123 | -2.0 | 0.0008 | Heme exporter subunit ; ATP-binding component of ABC superfamily |
| *Cell wall/membrane biogenesis* | | | | | |
| 700062 | *rffC* | 379 | -2.3 | 3.5E-07 | TDP-fucosamine acetyltransferase |

^a^FC is the fold change of the genes that exhibit significant (FC ≤ -2, false discovery rate (FDR) ≤ 0.005, minimum normalized read count = 10) differential expression. Only significant fold changes of genes non-discussed in our study are shown on this table for simplicity.

**^b^**Adjusted *p*-value for multiple testing with the Benjamini-Hochberg procedure which controls FDR.
