# Supplementary material for: Strand-specific transcriptomes of Enterohemorrhagic Escherichia coli in response to interactions with ground beef microbiota: interactions between microorganisms in raw meat
Source: BMC Genomics. 2017 Aug 3;18:574. doi: 10.1186/s12864-017-3957-2 (PMC5543532; doi:10.1186/s12864-017-3957-2)
Supplement: Supplementary file 6 — Discarded up-regulated genes in Escherichia coli O26:H11 21,765 in samples with microbiota compared to those without microbiota. (DOC 51 kb) [file 12864_2017_3957_MOESM6_ESM.doc]

Table S6: Discarded up-regulated genes in *Escherichia coli* O26:H11 21765 in samples with microbiota compared to those without microbiota

| Locus (ECO26H_v1_ #) | Gene name | Mean of normalized counts | FC^a^ | adj. *p*^b^ | Function or product | Identified genomes^c^ |
| --- | --- | --- | --- | --- | --- | --- |
| [270175](https://www.genoscope.cns.fr/agc/microscope/mage/getInfoLabel.php?id=22063419) | *ydcU* | 283 | 2.0 | 7.9E-05 | Putative spermidine/putrescine transporter subunit ; permease of ABC superfamily transporter | *S.* sp. S4 WGS APLA_V1_NZ_APLA ; *S. proteamaculans* 568 chr. Spro (83.9%) |
| [340040](https://www.genoscope.cns.fr/agc/microscope/mage/getInfoLabel.php?id=22063639) | *ydgT* | 1414 | 2.3 | 2.2E-07 | Post-transcriptional regulator | *S. fonticola* AU-AP2C WGS ASZA_V1 (75.8%) |
| [340076](https://www.genoscope.cns.fr/agc/microscope/mage/getInfoLabel.php?id=22063675) | *ydhC* | 334 | 2.1 | 0.0002 | Putative transporter; MFS superfamily | *S. liquefaciens* ATCC 27592 chr. M495_NC_021741 (64.2%) |
| [400095](https://www.genoscope.cns.fr/agc/microscope/mage/getInfoLabel.php?id=22064107) | *yodA* | 1329 | 2.0 | 1.8E-09 | Conserved hypothetical protein; putative metal-binding protein | *S. marcescens* FGI94 chr. D781 (67.6%); *Carnobacterium maltaromaticum* LMA28 chr. (54.2%) |
| [420014](https://www.genoscope.cns.fr/agc/microscope/mage/getInfoLabel.php?id=22064172) | *yeeF* | 791 | 2.0 | 1.5E-05 | Putative amino-acid/amine transporter | *S. fonticola* AU-AP2C WGS ASZA_V1 (79.7%)*; S.* sp. S4 WGS APLA_V1_NZ_APLA (78.7%) |
| [520036](https://www.genoscope.cns.fr/agc/microscope/mage/getInfoLabel.php?id=22065044) | *yqaB* | 647 | 2.0 | 4.5E-06 | Putative hydrolase | *S.* sp. S4 WGS APLA_V1_NZ_APLA ; *S. proteamaculans* 568 chr. Spro (72.3%) |
| [550018](https://www.genoscope.cns.fr/agc/microscope/mage/getInfoLabel.php?id=22065260) | *ssnA* | 77 | 2.5 | 0.001 | Putative chlorohydrolase  /aminohydrolase | *S. fonticola* AU-AP2C WGS ASZA_V1 (76.6%) |
| [60081](https://www.genoscope.cns.fr/agc/microscope/mage/getInfoLabel.php?id=22062065) | *_* | 156 | 2.1 | 5.7E-06 | Putative ribosome maturation protein | *S.* sp. S4 WGS APLA_V1_NZ_APLA (80.4%) |
| [60082](https://www.genoscope.cns.fr/agc/microscope/mage/getInfoLabel.php?id=22062066) | *ykgM* | 458 | 2.8 | 4.5E-16 | Putative ribosomal protein | *S. plymuthica* AS9 chr. SerAS9 (67.9%) |
| [710086](https://www.genoscope.cns.fr/agc/microscope/mage/getInfoLabel.php?id=22066325) | *emrD* | 1370 | 2.1 | 7.1E-05 | Multidrug efflux system protein | *S. fonticola* AU-AP2C WGS ASZA_V1 (72.6%) |
| [710126](https://www.genoscope.cns.fr/agc/microscope/mage/getInfoLabel.php?id=22066365) | *ligB* | 116 | 2.0 | 0.0006 | DNA ligase NAD(+)-dependent | *S. proteamaculans* 568 chr. Spro (50.6%) |
| [720029](https://www.genoscope.cns.fr/agc/microscope/mage/getInfoLabel.php?id=22066450) | *xylG* | 96 | 2.3 | 0.0001 | Fused D-xylose transporter subunits of ABC superfamily: ATP-binding components | *S.* sp. ATCC 39006 Chr. Ser 39006 (77.8%) |
| [p40034](https://www.genoscope.cns.fr/agc/microscope/mage/getInfoLabel.php?id=22067279) | *_* | 170 | 2.3 | 0.0004 | Putative eex protein | *S. symbiotica* Tucson WGS AENXv1 (50.7%) |
| [p50008](https://www.genoscope.cns.fr/agc/microscope/mage/getInfoLabel.php?id=22067296) | *istA* | 216 | 2.6 | 1.1E-08 | Transposase | *S. fonticola* AU-P3(3) WGS ASZB-v1 (91.5%) |

^a^FC is the fold change of the genes that exhibit significant (FC ≥ 2, false discovery rate (FDR) ≤ 0.005, minimum normalized read count = 10) differential expression. Only discarded genes are shown on this table. Expected genes to be differentially expressed due to the possibility of spurious alignment, and crosstalk between the reference genome and other similar genomes identified at genus level by 16S meta-genomic analysis, were discarded from this study.

**^b^**Adjusted *p*-value for multiple testing with the Benjamini-Hochberg procedure which controls FDR.

^c^Bacterial organisms identified at genus level by 16S meta-genomic analysis which may be similar to our reference genome. A score of nucleotide identity (between the altered gene and any other gene provided by GenBank and identified as being part of the genome sequencing data of the identified genus by 16S meta-genomic analysis) with more than 50% over 80% or more of their length was shown in parentheses.
