# Supplementary material for: Strand-specific transcriptomes of Enterohemorrhagic Escherichia coli in response to interactions with ground beef microbiota: interactions between microorganisms in raw meat
Source: BMC Genomics. 2017 Aug 3;18:574. doi: 10.1186/s12864-017-3957-2 (PMC5543532; doi:10.1186/s12864-017-3957-2)
Supplement: Supplementary file 7 — Others down-regulated genes in Escherichia coli O157:H7 EDL933 in samples with microbiota compared to those without microbiota. (DOC 36 kb) [file 12864_2017_3957_MOESM7_ESM.doc]

Table S7: Others down-regulated genes in *Escherichia coli* O157:H7 EDL933 in samples with microbiota compared to those without microbiota

| Locus  (Z #) | Gene name | Mean of normalized counts | FC^a^ | adj. *p*^b^ | Function or product |
| --- | --- | --- | --- | --- | --- |
| ECO571166 | _ | 42 | -2.5 | 0.0009 | Fragment of conserved hypothetical protein (partial) |
| 1966 | *_* | 70 | -2.0 | 0.004 | Putative ABC transporter periplasmic binding protein |
| 3199 | *_* | 343 | -2.1 | 0.0007 | Putative glycosyl transferase WbdP (Glycosyl transferase) |
| 4363 | *yqhC* | 396 | -2.0 | 7.1E-07 | Putative DNA-binding transcriptional regulator |
| 5408 | *yihL* | 331 | -2.3 | 8.0E-09 | Putative DNA-binding transcriptional regulator |

^a^FC is the fold change of the genes that exhibit significant (FC ≤ -2, false discovery rate (FDR) ≤ 0.005, minimum normalized read count = 10) differential expression. Only significant fold changes of genes non-discussed in our study are shown on this table.

**^b^**Adjusted *p*-value for multiple testing with the Benjamini-Hochberg procedure which controls FDR.
