# Supplementary material for: Strand-specific transcriptomes of Enterohemorrhagic Escherichia coli in response to interactions with ground beef microbiota: interactions between microorganisms in raw meat
Source: BMC Genomics. 2017 Aug 3;18:574. doi: 10.1186/s12864-017-3957-2 (PMC5543532; doi:10.1186/s12864-017-3957-2)
Supplement: Supplementary file 8 — Discarded up-regulated genes in Escherichia coli O157:H7 EDL933 in samples with microbiota compared to those without microbiota. (DOC 62 kb) [file 12864_2017_3957_MOESM8_ESM.doc]

Table S8: Discarded up-regulated genes in *Escherichia coli* O157:H7 EDL933 in samples with microbiota compared to those without microbiota

| Locus  (Z #) | Gene name | Mean of normalized counts | FC^a^ | adj. *p*^b^ | Function or product | Identified genomes^c^ |
| --- | --- | --- | --- | --- | --- | --- |
| [0520](https://www.genoscope.cns.fr/agc/microscope/mage/getInfoLabel.php?id=618691) | *pgpA* | 342 | 2.1 | 0.0008 | Phosphatidyl glycero phosphatase A | *Serratia plymuthica* AS9 chr. SerAS9 |
| [0559](https://www.genoscope.cns.fr/agc/microscope/mage/getInfoLabel.php?id=618706) | *mdlB* | 702 | 2.1 | 1.6E-06 | Putative fused ATPase and permease component of metabolite ABC transporter | *S. marcescens* FGI94 chr. D781 (81.8%) |
| [0958](https://www.genoscope.cns.fr/agc/microscope/mage/getInfoLabel.php?id=618863) | *_* | 170 | 2.0 | 4.2E-05 | Conserved hypothetical protein | *S. plymuthica* AS9 chr. SerAS9 |
| [1068](https://www.genoscope.cns.fr/agc/microscope/mage/getInfoLabel.php?id=621566) | *ybjG* | 506 | 2.1 | 0.0001 | Undecaprenyl pyrophosphate phosphatase | *S.* sp. S4 WGS APLA_V1_N_APLA; *S. proteamaculans* 568 chr. Spro (59.7%) |
| [2102](https://www.genoscope.cns.fr/agc/microscope/mage/getInfoLabel.php?id=619531) | *_* | 136 | 2.3 | 2.7E-05 | Conserved hypothetical protein | *S. marcescens* ww4 chr. Smww4 (55.93%) |
| [2479](https://www.genoscope.cns.fr/agc/microscope/mage/getInfoLabel.php?id=622090) | *pspC* | 179 | 3.2 | 8.4E-15 | DNA-binding transcriptional activator | *S. marcescens* ww4 chr. Smww4 (58.4%) |
| [2480](https://www.genoscope.cns.fr/agc/microscope/mage/getInfoLabel.php?id=622091) | *pspB* | 83 | 2.5 | 6.8E-05 | DNA-binding transcriptional regulator of psp operon | *S. marcescens* ww4 chr. Smww4 (82.2%) |
| [2482](https://www.genoscope.cns.fr/agc/microscope/mage/getInfoLabel.php?id=622092) | *pspA* | 1586 | 2.3 | 3.2E-08 | Regulatory protein for phage-shock-protein operon | *S. marcescens* ww4 chr. Smww4 (82.7%) |
| [2636](https://www.genoscope.cns.fr/agc/microscope/mage/getInfoLabel.php?id=619764) | *rsxC* | 535 | 2.0 | 2.6E-05 | Putative 4Fe-4S ferredoxin-type protein fused with unknown protein | *S. plymuthica* S13 NULL chr. 621 (67.11%); *S. marcescens* FGI94 chr. D781 (64.45%) |
| [2963](https://www.genoscope.cns.fr/agc/microscope/mage/getInfoLabel.php?id=619889) | *tyrP* | 378 | 2.0 | 0.0002 | Tyrosine transporter | *S.* sp. S4 WGS APLA_V1_NZ_APLA (80.4%); *S. proteamaculans* 568 chr. Spro (80.4%) |
| [3795](https://www.genoscope.cns.fr/agc/microscope/mage/getInfoLabel.php?id=622816) | *iscA* | 653 | 2.3 | 2.4E-07 | FeS cluster assembly protein | *S.* sp. S4 WGS APLA_V1_NZ_APLA (79.4%); *S. proteamaculans* 568 chr. Spro (79.4%) |
| [3796](https://www.genoscope.cns.fr/agc/microscope/mage/getInfoLabel.php?id=622817) | *iscU* | 758 | 2.0 | 1.8E-05 | Scaffold protein | *S.* sp. S4 WGS APLA_V1_NZ_APLA (92.2%); *S. proteamaculans* 568 chr. Spro (92.2%) |
| [3986](https://www.genoscope.cns.fr/agc/microscope/mage/getInfoLabel.php?id=620251) | *emrA* | 335 | 2.0 | 0.0007 | Multidrug efflux system | *S. fonticola* AU-AP2C WGS ASZA_V1 (70%) |
| [4107](https://www.genoscope.cns.fr/agc/microscope/mage/getInfoLabel.php?id=622976) | *truC* | 299 | 2.0 | 7.1E-06 | tRNA pseudouridine synthase | *S.* sp. S4 WGS APLA_V1_NZ_APLA; *S. proteamaculans* 568 chr. Spro (74.9%) |
| [4546](https://www.genoscope.cns.fr/agc/microscope/mage/getInfoLabel.php?id=623190) | *yhbE* | 1293 | 2.0 | 0.0001 | Conserved hypothetical protein; putative inner membrane protein | *S. marcescens* ww4 chr. Smww4 (79.5%) |
| [4620](https://www.genoscope.cns.fr/agc/microscope/mage/getInfoLabel.php?id=620526) | *dusB* | 7300 | 2.1 | 0.0002 | tRNA-dihydrouridine synthase B | *S.* sp. S4 WGS APLA_V1_NZ_APLA (87.5%) ; *S. marcescens* FGI94 chr. D781 (88.9%); |
| [4621](https://www.genoscope.cns.fr/agc/microscope/mage/getInfoLabel.php?id=620527) | *fis* | 1492 | 2.3 | 2.1E-06 | Global DNA-binding transcriptional dual regulator | *S.* sp. S4 WGS APLA_V1_NZ_APLA ; *S. marcescens* FGI94 chr. D781 (100%); |
| [5168](https://www.genoscope.cns.fr/agc/microscope/mage/getInfoLabel.php?id=620740) | *emrD* | 686 | 2.0 | 0.0006 | Multidrug efflux system protein | *S.* sp. S4 WGS APLA_V1_NZ_APLA ; *S. proteamaculans* 568 chr. Spro (72.1%) |
| [5648](https://www.genoscope.cns.fr/agc/microscope/mage/getInfoLabel.php?id=620966) | *yjbO* | 560 | 3.0 | 3.3E-18 | Putative membrane protein of unknown function | *S.* sp. ATCC 39006 Chr. Ser 39006 (71.7%) |
| [5782](https://www.genoscope.cns.fr/agc/microscope/mage/getInfoLabel.php?id=621020) | *hflC* | 1750 | 2.1 | 8.9E-08 | Modulator for HflB protease specific for phage lambda cII repressor | *S.* sp. S4 WGS APLA_V1_NZ_APLA ; *S. proteamaculans* 568 chr. Spro (82.9%) |
| [5853](https://www.genoscope.cns.fr/agc/microscope/mage/getInfoLabel.php?id=621064) | *mgtA* | 746 | 2.6 | 3.3E-07 | Magnesium transporter | *S. fonticola* AU-AP2C WGS ASZA_V1 (73.03%) |
| [5959](https://www.genoscope.cns.fr/agc/microscope/mage/getInfoLabel.php?id=623823) | *mdoB* | 615 | 2.3 | 1.7E-07 | Phosphoglycerol  transferase I | *S. marcescens* ww4 chr. Smww4 (55.2%) |
| [5974](https://www.genoscope.cns.fr/agc/microscope/mage/getInfoLabel.php?id=621115) | *rimI* | 255 | 2.0 | 4.6E-05 | Acetylase for 30S ribosomal subunit protein S18 | *S.* sp. S4 WGS APLA_V1_NZ_APLA (70.1%); *S. proteamaculans* 568 chr. Spro (70.8%) |

^a^FC is the fold change of the genes that exhibit significant (FC ≥ 2, false discovery rate (FDR) ≤ 0.005, minimum normalized read count = 10) differential expression. Only discarded genes are shown on this table. Expected genes to be differentially expressed due to the possibility of spurious alignment, and crosstalk between the reference genome and other similar genomes identified at genus level by 16S meta-genomic analysis, were discarded from this study.

**^b^**Adjusted *p*-value for multiple testing with the Benjamini-Hochberg procedure which controls FDR.

^c^Bacterial organisms identified at genus level by 16S meta-genomic analysis which may be similar to our reference genome. A score of nucleotide identity (between the altered gene and any other gene provided by GenBank and identified as being part of the genome sequencing data of the identified genus by 16S meta-genomic analysis) with more than 50% over 80% or more of their length was shown in parentheses.
